# Remarkable Menstruation

**Published:** 1879-12

**Authors:** 


Remarkable Menstruation.—Dr. Rodsewitch relates the following curious story: The widow of a peasant in the Province of Nijvi-Novgorod menstruated for the first time at the age of 36. She was married in her 15th year, without even having menstruated. From that time, and throughout all the years of her married life, she was continually either pregnant or nursing, and never saw her monthly periods. Her husband died when she was 36 years old, and her courses soon after appeared, and continued with great regularity. She had twins at the second, fourth and eighth confinement; so that she bore 16 children in all.—*Canada Med. and Surgical Jour.*

